# Address Delivered before the Vermont Association of Dental Surgeons, on the Advantages of Association

**Published:** 1855-01

**Authors:** Chapin A. Harris


### ARTICLE VIII.

*Address Delivered before the Vermont Association of Dental Surgeons, on the Advantages of Association.* October 25, 1854. By CHAPIN A. HARRIS, M. D., D. D. S.

MR. PRESIDENT AND GENTLEMEN
OF THE VERMONT ASSOCIATION OF DENTAL SURGEONS:

Honored by your invitation, I am here for the purpose of congratulating you on an occasion which is to constitute a new, and, I doubt not, a bright era in the history of dental surgery in the state of Vermont. Hitherto your efforts to enlarge your ability for usefulness, advance the cause of science, and elevate the dignity of your calling, have been isolated. You have depended, for the most part, each one upon his own individual experience, observation and research; and however industriously you may have applied yourselves to the cultivation of your peculiar department of physical alleviation, you have not, individually nor collectively, made the progress you would have done, had you all along enjoyed the advantage of frequent and free intercommunication with each other. Although you may have kept pace with the progress of the dental specialty of medicine, yet, neither you nor your professional brethren in other states of the union, and in other countries, have attained the height of excellence as practitioners and as men of science, which, both you and they, would have done, had the result of your united labors been, from time to time, given to each other and to the world.

In entering a liberal profession, every one who does it, incurs a debt that can only be cancelled by giving to his brethren whatever acquisition of knowledge he, with all his industry, may be able to make, and in doing so, he must not suppose he will be impoverishing himself. On the contrary, his contribution may serve as the basis, in the hands of others, of a still greater acquisition, or lead to some valuable discovery or improvement, the knowledge of which, coming back to him, may, to say nothing of the honor previously acquired by it, compensate him a thousand fold for the labor and trouble it may have cost him. Thus, without having deprived himself of any of his own individual resources, he confers a lasting and important benefit both upon his profession and upon mankind, while in return, he receives a full equivalent, with more than compound interest.

But this is a selfish view of the subject. It reflects no credit upon the donor. A liberal minded man is actuated by higher and nobler considerations. He gives for the sake of doing good to others, without reference to the benefit he may derive from it himself, and in doing so, he feels that he is only liquidating in part, a debt justly due from him to his professional brethren, knowing that without the knowledge previously received from the labors of others, he would not have been able to contribute any thing of value to the already accumulated stock of knowledge. Thus far, to use a commercial expression, he has been doing business upon borrowed capital—upon the wealth of professional knowledge which had been toilsomely elaborated by hundreds of others, who, perhaps, at the expense of comfort and ease, and without hope of any other reward than a desire to do good, had devoted the vigor and best energies of their lives to its accumulation. Having appropriated this knowledge to his own use, he is bound in honor to contribute to the utmost of his ability to the acquisitions previously made, and held, as common property, for the benefit of each and every member of his profession. The obligation resting upon him, cannot be canceled in any other way. Whatever discovery, or improvement in practice, he may make, belongs, according to all the recognized rules of etiquette, to the profession which numbers him among its members, because he receives from it the means which enables him to make it and use it.

Nor are his obligations to community at large at all less weighty. He owes as much to the latter as to the former. The prevention, alleviation and relief of human suffering, constituting his chief business of life, as well as the means of his support, if not of the acquisition of wealth, he owes it to his fellow man, to all who may seek his aid, confiding in his skill, to have at his command the entire resources of his art, to enable him most thoroughly to arrest the footsteps of disease and beat back the assaults of the destroyer. In assuming the responsibility of his calling, he virtually pledged himself both to it and to society, to labor diligently and faithfully to obtain the highest possible qualification for the performance of its duties. The pursuit to which he has dedicated himself, claims the active, constant and most persevering exercise of all his physical and intellectual energies. His time, therefore, is not his own, and he has no right to squander it away or permit it to run to waste; nor can he devote it to other objects of life without violating the most sacred implied pledges. He cannot live a life of inaction. He must labor early and labor late, and when not actually engaged in the exercise of the duties of his profession, he should be adding to his qualifications for them, either by study or by training his hands to the various manipulations they may be called upon to perform.

Thirty years ago, public opinion did not require that a dentist should be a man of scientific attainments. His profession was then regarded as a mere handicraft pursuit, requiring no knowledge of general medicine and collateral science. His duties were supposed to be confined to a few mechanical manipulations, which might be performed by almost any one possessing a little manual dexterity. But this opinion is rapidly giving way to a larger and juster appreciation of the importance of his specialty, and the diversity and extent of knowledge required to practice it successfully. An amount of preparatory study and instruction, equal to that required for a doctorate in medicine, is now beginning to be regarded as necessary to qualify a man to *commence* the practice of this department of medicine, and in proportion as this opinion has grown and diffused itself abroad, has scientific dentistry advanced, and its practitioners obtained cast in society and with the learned professions. A dentist of the present day, who has any regard for his own reputation, or the good of his patrons, feels it to be as necessary to be thoroughly acquainted with the standard and current literature of his profession, as well as with that of such of the collateral sciences as may be made subservient to skillful practice, as does the practitioner of general medicine, or any of its other specialties, to be well read in the various departments of the great healing art. With a well educated hand, and the knowledge thus obtained, he has at his command all the known resources of his calling, and it is only by the possession of them, that he can fully meet the claims which his professional brethren and society have upon him. The dentist who does not aspire to the position which such attainments are always certain to secure, deserves neither the respect of the one, nor the confidence of the other; and he who seeks it with the expectation of finding it in a day or week, will acknowledge, if he has high aspirations, long before he reaches the goal of his ambition, that

"The heights by great men reached and kept
Were not attained by sudden flight,
But they, while their companions slept,
Were toiling upwards in the night."—*Longfellow.*

But reading and exercising the hand in the various manipulations required in practice, are not the only means and sources of information which may be made available to the acquisition of professional knowledge. They are necessary—the one as much as the other, the former serving as a light to guide the latter, but much valuable additional knowledge may be obtained by frequent interchange of views and opinions between the members of the profession, and by united systematic investigation of subjects connected with those departments of science and art which come properly within their province. Every practitioner of common acumen, and possessing industry enough to think, must, necessarily, in the course of a year, accumulate many facts, the careful examination and comparison of which, may lead to important and useful practical results. There is no way by which such facts can be better collected, and made available, than by the means which you have adopted, and I hail the organization of this association as auspicious to the advancement of scientific dentistry in the United States, where, for the last quarter of a century, it has probably progressed more rapidly than in any other part of the world. Actuated by a spirit of noble emulation, laying aside whatever feelings of jealousy you may have heretofore entertained towards each other, you meet as men engaged in a common pursuit, for the promotion of a common object, the elevation of the dignity of your profession and the good of your fellow men. You have this day erected an altar, dedicated to science, before which, you plight your vows of truthful devotion, unitedly resolving to devote the best energies of your lives to the advancement of the pursuit in which you are engaged.

Gentlemen, I have not language to express the gratification I experience in being with you on this occasion. Although but a few hours were all the leisure I could obtain from arduous professional occupations, to prepare for the performance of the duty to which you, in your kind partiality, were pleased to invite me, and although at the expense of much personal comfort, I could not consent to forego the anticipated pleasure of being with you at this time, feeling, as I have always done, the liveliest interest in every movement which has for its object the advancement of a branch of the curative art, to the study and practice of which, nearly my whole life has been devoted. But in accepting the invitation with which you honored me, I had another object in view. It was, to make the acquaintance, and take by the hand, my professional brethren of the Green Mountain State. I also desired to gain instruction, for I always expect to be a student, trusting I shall never be too old nor too proud to learn, from your deliberations, that in returning to my own distant field of labor, I might do so, encouraged and strengthened, by the knowledge I should obtain, in the arduous path of duty.

So manifest are the benefits of association, that it scarcely seems necessary to offer an argument to prove a fact, which the history of the past has already so fully established. Through the agency of association, the strength of numbers is brought to bear upon any given object or amelioration we desire to accomplish; and through this agency, nearly all the great enterprizes of the world, whether for the promotion of the cause of benevolence, or science, or art, or the agricultural interests, have been brought into successful operation. By this means, the aggregate wisdom of immense masses of mind, has been scattered broadcast over the world, and millions upon millions of its inhabitants have been enlightened and profited thereby.

The amount of good which has resulted from association, is incalculable, and yet, the numerous scientific, literary and benevolent institutions, now the praise and wonder of the world, had their origin in small beginnings. The French Academy, which has grown to almost gigantic strength, originated in a private meeting of a few literary gentlemen of Paris, who met, on a fixed day, once a week, for the purposes of conversation, and to criticise each other's works. By the unprejudiced and friendly strictures thus elicited, the works, to the gratification of the authors, were, in most instances, improved. The existence of this literary senate having, a few years after, been communicated to the French minister, Cardinal Richelieu, he, thinking it might be made to redound to his own glory, had it constituted a public body by letters patent, permitting the originators however to frame their own constitution, and elect their own officers. Letters and science are nearly a century in advance, of what they would have been but for the aid and encouragement they have received from this great republic of literature. The Royal Society of England originated in pretty nearly the same way, and it is well known that science and art are indebted to the encouragement and aid of this illustrious association for some of the most important and valuable contributions they have ever received. It has thus conferred inestimable benefit upon mankind. To the London Society of Arts, established in 1754, the world is largely indebted. Through the liberal encouragement of this noble institution, many useful inventions, which otherwise would never have been known, have been rendered available to the great practical purposes of life. The premiums alone, distributed by it in about ninety years, amount to more than half a million of dollars. The appointment of Professor Richard Owen, by the Royal College of Surgeons, to the lectureship of the Hunterian Museum, has secured to dental science, as well as to comparative anatomy, some of their richest and most valuable contributions. In his Odontography alone, which, but for this appointment, would never in all probability have appeared, Professor Owen has erected for himself a far more splendid and enduring monument of fame, than any that could have been chiseled from the costliest marble. As valuable as this great work is to the odontologist and comparative anatomist, it is equally so to the zoologist, and paleontologist. Each of the sections of the British Association has also contributed its full quota to its own particular department of science.

But we need not go abroad for examples of the good that has been effected through the agency of association. We have them on every side, and all around us in our country. The American Medical Association, though still in its infancy, is throwing out in its annual transactions, contributions as rich in scientific and practical value as have been furnished by any similar organization in the old world. The science of medicine in all its departments, has also received valuable accessions to its literature from the published transactions of the various state, city and county medical societies of the union, which have, from year to year, been given to the profession. The Philadelphia, New York, and Boston Academies of Natural Science, have done much to augment the general stock of knowledge belonging to the peculiar departments, over which they have extended their fostering care. The light and knowledge sent to the various heathen nations and tribes of the earth, through the direct and active instrumentality of the benevolent institutions of our country, have contributed, in an eminent degree, to ameliorate the condition of the inhabitants of many of the dark and benighted portions of the world.

But it is not necessary to enter into detail: it would require volumes to enumerate the benefits which have resulted from association; I have merely adverted, in a general way, to a few of the results which have been produced by the co-operation of numbers, directed to the accomplishment of certain given objects, with a view of illustrating the advantages to be derived from organizations of this kind. The experiments of association in our own profession, though few and of recent origin, are, nevertheless, sufficiently encouraging to warrant the belief, that they may be made to contribute immensely to the advancement of the science and art of dental surgery. Although they may not, as yet, have realized the expectations of all, still, it cannot be denied that they have been productive of much good. Our literature has been enriched by some of the published transactions of these associations, and we have also derived valuable practical information from them; they have awakened too, a spirit of inquiry and emulation among the members of the profession generally, which, even now, is producing the most salutary and beneficial effects. Dentists begin to feel that they must be men of science, as well as mechanics, and every day the field of their researches and investigations is becoming more and more enlarged; they begin to realize, even in reference to dentistry, what an anonymous writer says, in speaking of Lord Bacon's prophecies of the advance of science, that "Knowledge partakes of infinity," that it widens with our capacities; the higher we mount in it, the vaster and more magnificient are the prospects it stretches out before us. Nor are we in these days, as men are ever apt to imagine of their own times, approaching to the end of them; nor shall we be nearer the end a thousand years hence than we are now. The family of science has multiplied; new sciences hitherto unnamed, unthought of, have arisen. The seed which Bacon sowed, sprung up, and grew to be a mighty tree, and the thoughts of thousands of men came and lodged in its branches, and those branches spread so broad and long, that in the ground the bended twigs took root, and daughters grew about the mother tree, a pillared shade high over-arched and echoing walks between walks, where poetry may wander and wreath her blossoms around the mossy stems, and where religion may hymn the praises of that wisdom, of which science exacts the hundred aisled temple.

These remarks may be made to apply with as much force to the science of dentistry, as to science in general. Every new discovery, and every new triumph of skill, encourage the practitioner to push on his researches, to extend his explorations into more distant regions, and to attempt still greater and more formidable achievements. Thus, from a mere mechanical art, which could be acquired in a few days or weeks at most, it has grown to be a science, requiring for its acquisition years of the most persevering application. So it must ever continue to progress, its advance all the while becoming more and more rapid, and among the agencies which are to contribute to this, association will play a prominent part.

In entering into the spirit of union of purpose, you do so for the accomplishment of a great and general good, to bring to the highest perfection of influence and usefulness, the profession of which you are members. Having entered into the bonds of association, let me say to you in the language of entreaty, for I would fain cherish high expectations with regard to the benefits that shall result from your labors; cultivate feelings of the warmest friendship, and mutual respect for each other. In all your meetings, and in your social and professional intercourse, carry with you a generous and conciliating spirit of self-denial, carefully avoiding all causes of dissension and dispute, assured, that if discord get among you, or the demon envy take his seat in your midst, the strong cords of brotherly love will be broken, and there will be an end to your usefulness, and all your hopes of well earned fame will be scattered like mists of morning before the rising sun. Reproach would settle down upon your entire society, which the labor of years would not be able to remove; and, in proportion as your association would be brought into discredit, would each individual member share in the loss of respect and general consideration sustained by it. These considerations, should make you jealous of each other's reputation, and of the character you may acquire in your collective and associated capacity.

If it would not be thought presumption in one who is a stranger to nearly every member of this society, I would throw out, for your consideration, a few simple suggestions with regard to the best means of carrying out the great purposes of your association. Although, what I may say upon this subject, may already have claimed your attention, and been determined on, still, the importance of it cannot be too strongly urged.

If you would have the transactions of each meeting, rich in scientific contributions and practical information, assign to committees or members appointed for the purpose at each convocation, the investigation of certain specified subjects, with instructions to report the result of their researches at the next succeeding meeting. By this means, if the committees acting under such appointments, shall be properly impressed with the importance of executing to the best of their ability the trust committed to their care, the archives of your society, will, in a short time, become enriched with a vast amount of interesting and instructive knowledge. Thus your association, conducted in peace and harmony, will become emphatically a school of dentistry, in which each member will be both teacher and learner, communicating and receiving instruction, and profiting by all the knowledge, that shall, from year to year, accumulate at a common centre.

Scientific associations, as national governments, should be conducted upon simple principles, in accordance with rules easily understood, and rigidly enforced; and individuals entering into a voluntary compact, for the accomplishment of any given object, without a fixed determination to comply with all its requisitions, have no right to expect that the beneficial purposes for which it was made, will ever be realized. It is no honor to any one to be a member of a society which contributes nothing to the advancement of science, or to the promotion of some general good; and this can only be done as the operations of the association are properly directed, and its rules and regulations carried into full force and effect by the active co-operation of its members.

As the honor of our common profession will be concerned in your doings, let it not be considered presumption on the part of your speaker, to say, that in assuming the position you now occupy, it will be your duty to discountenance quackery in every shape in which the protean monster may present his worthless claims to public consideration. But while you do this, be ever ready to extend a helping and encouraging hand to aspiring merit. Even though the subject of it may not have reached the summit of professional excellence, still if he is urging himself on in the road which leads to it, aid him by advice or otherwise, as the circumstances of the case may require, and as you have ability, in his onward and upward course. You may thus make a lasting friend, and add a bright ornament to the profession. The ardent energies of many a worthy industrious young man, have been crushed and paralyzed in the bud, before they had time to blossom or bring forth fruit, for want of a little timely aid, and friendly advice.

Hitherto I have done little more than suggest the mode in which associations may be made conducive to the increase of individual knowledge and improvement in personal skill, and to the augmentation of that common stock of experience and thought which constitutes the science of our profession. Viewed only in this respect, we cannot doubt the probability of great advantage through this organization to yourselves severally, to dental surgery, and to the community whose ministers we are.

But, gentlemen, there is another and even more grateful aspect in which we may regard efforts of this character, when turning away from the more direct and material consequences, to be anticipated from such reunions, we consider the social and moral results necessarily flowing from them.

That God created of one blood all the dwellers in the earth, to whom he has given to bear his image, is a truth, proclaimed by the laws of human life, as distinctly as by the voice of inspiration. It is useless for self styled philosophers to try to argue us out of our consanguinity. The affinities of our kind lie deeper than the skin: the ultimate links of generic unity, are not fastened to cranial protuberances, nor turned around facial angles. The inscrutable inward life, defies mathematical measurements, and its unschooled instincts follow their sympathetic bent, utterly heedless of the recent canons of dogmatic physiology. He who studies man without considering the soul that is in him, may learn something of the box, but may not pretend to know the qualities of the jewel. Man, the intelligent, the moral, the immortal spirit, is not to be measured by compasses, nor scrutinized by microscopes, nor classified by mummy peelers. Let them stick to their dried beetles, and hieroglyphics, and male priestesses. Man, the soul, must be studied by men with souls, living, fervent souls, pursuing knowledge with some other senses than the corporeal ones, and recognizing other facts than the phenomena of matter.

We are one family, and the perfection of our race is to be accomplished through the full, complete, and eternal recognition of this great fact. The practical evil of our present condition is, that we are separated from one another. Sin has exploded on the domestic hearth and broken up the family circle. Selfishness has isolated every man from his fellow, and during the long dreary years of our evil history, we have been struggling to bring what we may of happiness within our own little world and secure it from the grasp of others as eager and as self-limited in their purposes as ourselves. Had solitary satisfaction been possible, and the means of gratification within the compass of individial powers, the whole race would have been broken up into disconnected and hostile units, and all that is affectionate in the nature of man must have withered and died. But the wisdom of the Creator had by the limits and variation of individual natures provided against such complition of selfish life. Necessity compels fraternity. A law of dependence subjugating all wills compels perpetual intercourse and interchange of benefits. The monarch is but a man with wants common to the lowest, and whatever his conventional superiority, his personal dependence is upon the obscure and ignoble. David could gather money to build the temple, and Solomon could arrange for its construction, but without the aid of an unknown man from Tyre, the glorious conception would have been an abortion, a jumble of artistic failures. The comfort of Alexander the Great, and of Homer, and Milton depended no little upon the successful labors of their cooks. Monarchy and literary reputation would be dreaded and dismal elevations were they necessarily connected with the penalty of bad dinners. We are necessary to one another. Some have mind and some have muscle. The timid lean upon the bold, and the bold upon the prudent, and the prudent upon the wise, and the wise upon the materials gathered by the patient and self-denying. In all the walks of life, the material necessities of men compel them to walk together: if wolves, they hunt in packs; if sheep, they lay their fleeces together; gather warmth from contact and quiet timidity by aggregation. Selfishness itself drives us together and compels confederation. All this, however, would of itself do nothing for the moral advantage of the race, were it not for the fact, that there are latent sympathies in the human breast, which exercise their affinities within near distances, and spring into active movement from mere propinquity to our kind. I can neither explain nor describe that singular warming about the heart, that unwonted benevolence of feeling, that tender sentiment of charity that is begotten to some extent even in the coldest natures, by the mere association of human beings.

Every one has noticed the different feelings to his fellow passengers with which he got into a stage-coach and got out of it. On entering, there is a concentrated purpose to take care of yourself; a snarling of the heart over comfortable places; an ill-natured survey of opposite physiognomy and encroaching angular projections; a most uncharitable classification of the human specimens about you as to social and moral distinctions. But a little shaking together develops very different sensations. The jolting of the vehicle that almost breaks your bones, quite breaks the ice about your heart. A good natured smile from your neighbor thaws it. Kindly affections bubble from unknown depths of the spirit. You are suddenly seized with a determination to sacrifice your comfort to that of somebody else, but somebody else feels the same heroic disposition towards you. Conversation becomes animated and extraordinarily smart. You are surprized at your own wit, and wonderfully with that of others. Positive evils become under the witchery of association, incidental provocations of good. Every dig of the angular elbow of the lean man opens a fountain of charity, and the ponderous lurches of your fat neighbor, squeeze out of your heart rich drops of the oil of human kindness. Should you be favored with an upset you are friends for life. Should one of the party, so suspiciously scrutinized at the commencement of acquaintance be a young lady and you a young man, the chances are that you twain may cleave together with such tenacity as in after life to be reckoned a unit.

Now, what is all this but the awakening of the old family feeling, which so long dormant, yet lives and responds to the sympathetic affinities, when once more brought into the sphere where it was destined to be the mainspring of human action.

I need not remind you, gentlemen, that the whole scheme of our beautiful christianity is based upon the fundamental idea of the human race, and that its avowed purpose is to reunite us, a holy family, whose bond shall be the common paternity of God, whose law shall be the spontaneous decisions of love, and whose life, pure benevolence.

Now, gentlemen, your coming together will do you good. It will thaw your hearts. It will interrupt the ordinary selfish routine of thought. It will correct wrong and uncharitable opinions of one another. It will provide you with friendship, and oil the dry and dusty wheels of life. While it corrects the hasty and unjust opinions of one towards his neighbor; it will equally correct the opinions of his neighbor towards him; so that while you are dismissing enmities you will be disarming enemies.

In the social advantages, then, of the organization now effected, I contemplate not the least important and interesting of its future benefits, and from my heart I rejoice in every event which promises to make operative, in any degree, the heaven-descended precept, "love one another."

That this association may accomplish the purposes of its projectors; that it may procure and preserve the now scattered facts of individual experience; that it may inspire hope of excellence and inspirit effort to attain it; that it may elevate our profession, by making tributary to it wealth of science and dignity of character; above all, that it may exert a benign influence upon the heart, and through the amenity of intercourse draw you closer to each other and to mankind, these are the sincere wishes and not unreasonable hopes of your unworthy speaker.

